# Factors associated with high prevalence of PfCRT K76T mutation in *Plasmodium falciparum* isolates in a rural and urban community of Ogun State, Nigeria

**Published:** 2017-08-01

**Authors:** Olajoju T. Soniran, Olufunmilayo A. Idowu, Segun S. Ogundapo

**Affiliations:** 1 Biology Research Unit, Akanu Ibiam Federal Polytechnic, Unwana, Ebonyi State, Nigeria; 2 Pure and Applied Zoology Department, Federal University of Agriculture, Abeokuta, Ogun State, Nigeria; 3 Biochemistry Research Unit, Akanu Ibiam Federal Polytechnic, Unwana, Ebonyi State, Nigeria

## Abstract

**Background:**

Antimalarial drug-resistant *Plasmodium falciparum* strains have been a major obstacle to the global efforts of controlling and eliminating malaria. The hope of reintroducing chloroquine for the treatment of uncomplicated malaria follows recent reports on decreases in the prevalence of chloroquine-resistant *P. falciparum* in several countries and recently, its total disappearance in Malawi and Zambia. In Nigeria, the discontinued use of chloroquine for malaria treatment was officially announced in 2005. A few available reports have shown a persistent high prevalence of the major biomarker of chloroquine resistance in southwest Nigeria. However, information on its prevalence in rural and urban areas is scanty. We investigated possible factors associated with the prevalence of a biomarker for chloroquine-resistance in Ogun State, southwest Nigeria.

**Materials and methods:**

Parasite DNA was extracted from dried blood spots collected by finger-prick in malaria symptomatic and asymptomatic subjects attending the urban-based State General Hospital and a rural-based Primary Health Centre. A structured questionnaire was used to collect data on malaria/fever treatment history. Nested Polymerase Chain Reaction (PCR) followed by Restriction Fragment Length Polymorphisms (RFLP) analysis was used to detect mutations in the *P. falciparum* chloroquine resistance transporter (*Pfcrt*).

**Results:**

Of the 243 participants recruited for this study, 56 were found to harbour *P. falciparum* parasites, of which 62.5% (35/56) showed symptoms of malaria. Prevalence of *P. falciparum* chloroquine-resistant strains (*Pfcrt* K76T) was 69.6%. The prevalence of *Pfcrt* K76T recorded in the rural area (91.7%) was significantly higher (P<0.05) than that in the urban area (53.1%). There was no correlation between prevalence of chloroquine-resistant strains and malaria symptoms in the rural area. However, prevalence of chloroquine-resistant strains was significantly higher in malaria-symptomatic subjects from the urban area.

**Conclusions:**

Drug-resistant *P. falciparum* strains recorded in the rural area were associated with self-medication and patronage of drug vendors who continue to sell chloroquine. These findings present the importance of continuous surveillance of biomarkers indicating drug resistance especially now that antimalarial drug resistance is a threat to malaria eradication.

## 1 Introduction

Malaria remains a disease with devastating global impact, killing more than 800 thousand people every year; the vast majority of these children below the age of five [1]. Malaria induced by *Plasmodium falciparum* is responsible for a high percentage of morbidity and mortality recorded in sub-Saharan countries, including Nigeria. One of the major threats to malaria control is the spread of drug-resistant parasites.

The rise of antimalarial drug resistance has dominated global malaria control programmes since resistance to chloroquine was first documented in patients in 1959 [2,3]. Chloroquine (CQ) was officially withdrawn in Nigeria as the first-line antimalarial drug in 2005 because of widespread and high-level clinical failure rates across the country [4]; the use of sulphadoxine-pyremethamine (SP) was subsequently adopted. In other parts of the world, SP was also adopted as the recommended first-line therapy for uncomplicated falciparum malaria because of high prevalence of chloroquine resistance. However by the 1980s the clinical efficacy of SP reduced in SE Asia partly because of its extensive usage and reputation as a single regimen that was safe, affordable and effective. Hence, alternative treatment regimens had to be deployed. Presently, the official first-line antimalarial drug recommended by WHO is artemisinin -based combination therapy (ACT). However, suspected decreased susceptibility to ACT or, at least, longer parasite clearance times have been described in Cambodia and this was reported to have a genetic basis [5–9].

The tools of molecular biology have had a major impact on the understanding of SP resistance. Molecular analysis of parasites could be achieved by polymerase chain reaction (PCR) to amplify the small amount of parasite DNA in a spot of blood from an infected person and simple analytical procedures were designed to identify the relevant genetic changes.

Chloroquine treatment failure in *P. falciparum* infection is significantly associated with a point mutation in the *P. falciparum* CQ-resistant transporter gene (*Pfcrt*) and this could therefore be used as a population marker for CQ resistance in Nigeria [10,11]. The replacement of lysine with threonine at position 76 (K76T) is important for the observation of *in vitro* CQ resistance [12]. It has been suggested that reducing the use of CQ in a region could result in the re -emergence of CQ-sensitive *P. falciparum,* thus permitting the reintroduction of this safe and affordable drug [10]. The return of CQ-susceptible malaria has been reported in many countries, but a complete disappearance of CQ resistance has only been observed in Malawi and Zambia [12].

Very high prevalence (96.9%) of *Pfcrt* K76T mutation was reported in *P. falciparum*-positive blood samples collected from children at the University Teaching Hospital, Sagamu, Ogun State [4] while 48% prevalence of *Pfcrt* (K76T) in *P. falciparum* isolates from Ogun State was reported in another study [13]. A previous study had earlier reported on the pattern of rural-urban acquisition of the *Pfcrt-T76* allele in Lagos State, Nigeria. A molecular surveillance for *Pfcrt* T76 alleles of CQ-resistant strains in children with acute uncomplicated *falciparum* malaria showed a rural-urban *PfcrtT76* acquisition of 48.7 vs. 73.7% and 67.3 vs. 74.6% due to monoclonal and polyclonal *P. falciparum* parasitaemia, respectively, suggesting unstable but increasing prevalence of *PfcrtT76* allele acquisition in the rural area [14].

This study was designed to evaluate the factors associated with the prevalence of *PfcrtK76T* allele in samples of subjects presenting acute uncomplicated *P. falciparum* at a rural-based Primary Health facility in Odeda Local Government Area and an urban-based State General Hospital in Abeokuta, south L.G.A. of Ogun State.

## 2 Materials and methods

### 2.1 Study population and sample collection

Two hundred and forty three (243) subjects aged 3-75 yrs, 63.0% and 37.0% of the subjects being symptomatic and asymptomatic for malaria, respectively, were enrolled in the study. The cohorts were recruited from a Primary Health Care Centre in Baale Ogunbayo village (n= 58; male/female=5/53), a rural settlement, located in Odeda L.G.A. and State General Hospital (n=185; male/female=76/109), located in urban Abeokuta, Abeokuta South L.G.A. Observation showed that in the rural community, women seek medical attention more frequently than men. Samples were collected between February 2013 and March 2014 during peak malaria transmission seasons (February to March and May to September).

Blood samples were collected from both symptomatic and asymptomatic individuals visiting the urban/rural facilities. About 2-5 ml of blood was drawn (venipuncture) with a sterile disposable syringe and transferred to a heparinised centrifuge tube. Rapid diagnostic test (RDT) detecting HRP-2 (*P. falciparum* only) was used to screen for *P. falciparum*-positive blood samples. Malaria-symptomatic patients were referred to a doctor. Dried blood spots were prepared on 1.5 x 7.0 cm strips of Whatman (Brentford, United Kingdom) 3MM filter paper for PCR analysis. The strips were kept in plastic bags containing small packets of silica gel as desiccants until use.

### 2.2 Molecular analysis and genomic sequencing

Extraction of *P. falciparum* genomic DNA was performed according to the method of Olukosi *et al.* [14], with few modifications. 1 μl aliquot of the extracted genomic DNA was used as template for nested PCR in a primary reaction volume of 25 μl to generate a primary fragment (outer *Pfcrt* locus = 206 bp) that was subsequently used as a template (1.0 μl) for a secondary PCR to generate a secondary fragment (inner *Pfcrt* locus = 145bp) [15]. Two sets of primers for PCR and allele-specific restriction analysis (ASRA) of *Pfcrt* codon 76 [15] were used. For primary PCR, the sense primer was 76-A (5'GCGCGCGCATGGCTCACGTTTAGGTGGAG3') and the antisense primer 76-B was (5'GGGCCCGGCGGATGTTACAAAACTATAGTTACC3'). The primary PCR components, in a final volume of 25 μl, were 22 μl of Supermix (invitrogen, USA), 0.5 μl of each primer (primer ‘A’ and primer ‘B’), 1 μl of RNA-free water, and 1 μl of DNA template. The cycling protocol was as follows: 95°C for 5 min for initial denaturation; 40 cycles of 94°C for 30 s, 52°C for 40 s and 72°C for 30 s; and a final extension of 72°C for 5 min. This was followed by the use of primers 76-D1 (5'TGTGCTCATGTGTTTAAACTT3') and 76-D2 (5'CAAAACTATAGTTACCAATTTTG3') for secondary PCR of 25 cycles as follows: 94°C for 30 s, 52°C for 40 s and 72°C for 30 s; and a final extension of 72°C for 5 min.

The resulting PCR products (10 μl) were electrophoresed on a 2% agarose gel, stained with ethidium bromide (5 μg/ml) and their molecular weight determined by extrapolation using a 100-bp ladder of molecular weight markers (Invitogen Life Technologies, USA) under ultraviolet transillumination.

5 μl of secondary fragment representing the inner *Pfcrt* locus (size = 145 bp) was digested for 5 hrs with 0.5 μl *Apo* I (New England Biolabs, Beverly, MA) at 50^0^C in a total 20 μl reaction using New England Biolabs #3.1 buffer. Digestion to 99 and 46 bp fragments indicates the wild type *Pfcrt* allele with lysine (K) encoded by codon 76 of the gene, while the mutant *Pfcrt* allele with threonine (T) at position 76 is uncut by the restriction enzyme [15]. *Pfcrt* alleles from *P. falciparum* DD2 (mutant) and HB3 (wild type) strains were used as controls, respectively.

The service of Macrogen, USA, was solicited for sequencing of PCR products to confirm likely mutations dictating drug sensitive and resistant strains of *P. falciparum*. A total of 8 randomly picked samples (four K76 samples and four T76 samples from the study areas) were sequenced to validate the result from PCR typing of the *Pfcrt* locus. Sequencing reactions were performed using BigDye® v3.1 (Life Technologies, Applied Biosystems) following the manufacturer’s protocol. Sequence detection was performed by capillary electrophoresis on a 3730xl Genetic analyser (Life Technologies, Applied Biosystems) using a 50 cm array, the Long DNA sequencing module (LongSeq50_POP7) and the KB analysis protocol (KB base-caller) with the default instrument settings. Post-detection, raw signal data was initially processed on the 3730xl Genetic analyser computer using Sequencing analysis v5.3.1 (Life Technologies, Applied Biosystems).

### 2.3 Data analysis

Data were presented as proportions and stratified by data on malaria/fever treatment. Disparity between proportions was evaluated using the STATCALC programme of Epi-Info version 6 software (CDC, Atlanta, GA).

### 2.4 Ethical clearance

Ethical approval (OOUTH/DA 326/451) for the study was obtained from the State Ministry of Health, Ogun State, through the Health Research Ethics Committee, Olabisi Onabanjo University Teaching Hospital. Approval was also sought from the medical directors and administrators of the facilities before sample collection. Oral informed consent was obtained from each participant. Anonymity of the study subjects was strictly enforced. Samples were collected and handled solely by trained medical laboratory staff. The study posed no risk to participants except for the transient pain they felt during blood collection. Sterile techniques and disposable, single use materials were used throughout the study period.

## 3 Results

**Figure 1:**
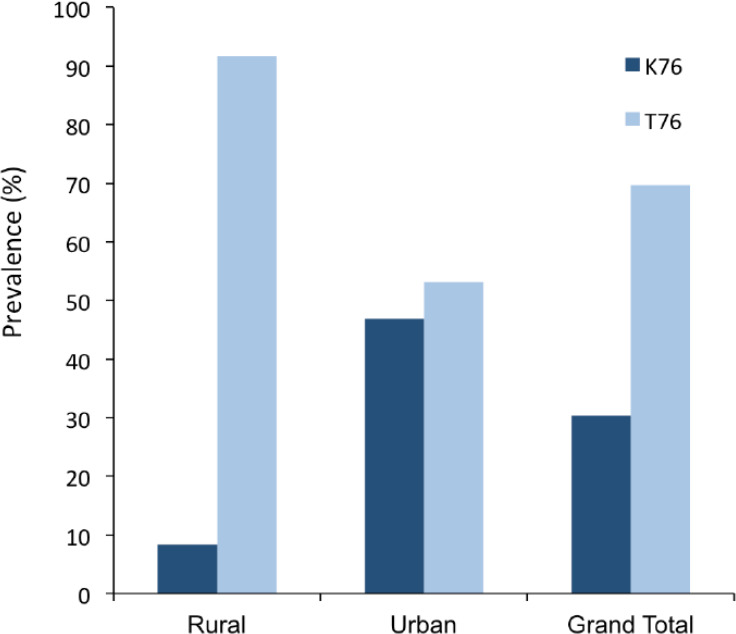
Prevalence (%) of *Pfcrt* K76T in the rural area and urban area.

Of the 243 subjects screened for *P. falciparum*, PCR confirmed 56 (23.1%) positive for parasite DNA; 32 (17.3%) samples from the urban area and 24 (41.4%) from the rural area. Overall, the prevalence of the *Pfcrt*T76 mutant allele was 69.6% (39/56) and that of the K76 wild-type allele was 30.4% (17/56). In the rural area, 91.7% (22/24) had the T76 mutant allele whereas in the urban area 53.1% (17/32) had the mutant allele; this difference was significant (P=0.002) (Figure 1).

**Figure 2:**
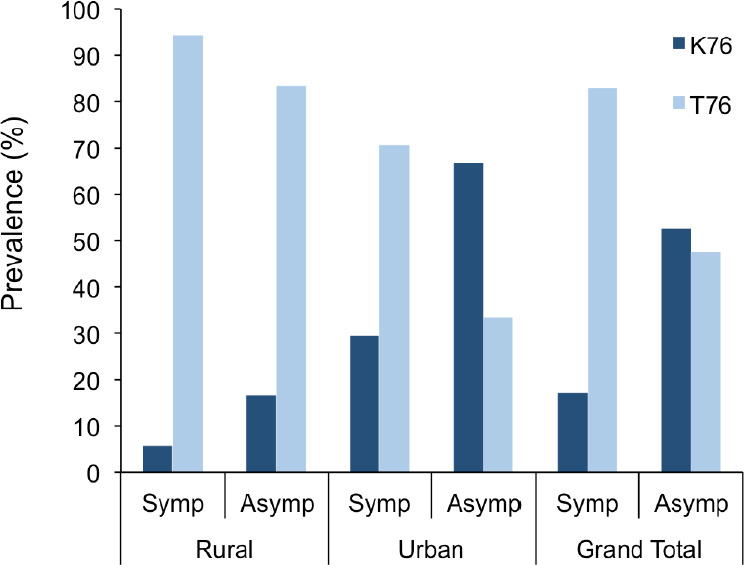
Prevalence (%) of *Pfcrt* K76T and malaria symptoms.

Of the 35 malaria symptomatic subjects 82.9% had the T76 mutant allele while 47.6% of the 21 asymptomatic subjects had the mutant allele. This difference in prevalence of *Pfcrt*T76 mutant allele between malaria symptomatic subjects and malaria asymptomatic subjects was significant (P=0.01). In the urban area, 70.6% of the symptomatic subjects and 33.3% of the asymptomatic subjects expressed the T76 mutant allele. This difference was also significant (P=0.04). However, in the rural area 94.4% of the symptomatic subjects and 83.3% of the asymptomatic subjects displayed the T76 mutant allele. This difference was not significant (P=0.40; Figure 2).

Medications frequently used were herbs only, herbs combined with antimalarial drugs, or antimalarial drugs only (Table 1). From the total samples expressing the T76 mutant allele, 10 (25.6%) subjects used herbs only, 6 (15.4%) combined herbs with antimalarial medicine, 15 (38.5%) used antimalarial drugs only while 8 (20.5%) did not recollect medication often used. In the group using herbs only, 8 (36.4%) and 2 (11.8%) subjects expressed the T76 allele in the rural and urban areas, respectively. In the group combining herbs and antimalarial drugs this was 4 (18.2%) and 2 (11.8%), respectively; in the group using antimalarial drug only, 8 (36.4%) and 7 (41.2%), respectively. In the group that did not recollect medication often used, 2 (9.1%) and 6 (35.3%) subjects expressed the T76 allele in the rural and urban areas, respectively. The difference in the proportion of T76 alleles expressed in the last group (no idea of medication) between the two study sites was significant (P=0.04).

**Table 1:** Medication frequently used by subjects harbouring the K76 and T76 Pfcrt alleles in the two communities.

	Rural			Urban				Grand Total		
Medication used	n	K76	T76	n	K76	T76	P-value	n	K76	T76
Herbs only	9	1 (11.1)	8 (88.9)	2	0 (0)	2 (100)		11	1 (9.1)	10 (90.9)
			(36.4)*			(11.8)*	0.08*			(25.6)*
Herbs + drugs	4	0 (0)	4 (100)	6	4 (66.67)	2 (33.33)		10	4 (40)	6 (60)
			(18.2)*			(11.8)*	0.58*			(15.4)*
Drugs only	8	0 (0)	8 (100)	13	6 (46.15)	7 (53.85)		21	6 (28.57)	15 (71.43)
			(36.4)*			(41.2)*	0.76*			(38.5)*
Not known	3	1 (33.33)	2 (66.67)	11	5 (45.45)	6 (54.55)		14	6 (42.86)	8 (57.14)
			(9.1)*			(35.3)*	0.04*			(20.5)*
Total	24	2 (8.33)	22 (91.7)	32	15 (46.88)	17 (53.1)		56	17 (30.36)	39 (69.6)

n: number examined; ()*: proportions across column; P-value*: probability value between proportions of T76 in the rural area and urban area.

Overall, 55.4% (31/56) of the subjects reported to use antimalarial drugs frequently. Twenty-two subjects (71.0%) of those using antimalarial medicine expressed the T76 mutant allele. Out of these 22 subjects the proportion that claimed to have used ACT, artesunate, sulfadoxine pyremethamine (SP) and ‘no idea of medication used’ were 7 (31.8%), 2(9.1%), 1(4.6%) and 12(54.6%), respectively (Table 2). In the rural area, 13 (92.9%) of those using antimalarial drugs expressed the T76 allele. A higher percentage (92.9%) of these subjects had no idea of the brand of antimalarial medicine used in the last malaria treatment while 7.1% reported to have used ACT for malaria treatment. In the urban area, 9 (52.9%) of those using antimalarial drugs expressed the T76 allele. A higher percentage (66.7%) of these subjects reported to have used ACT, while 22.2% and 11.1% reported using artesunate and SP, respectively. Difference in the prevalence of the T76 allele among subjects without any idea of the antimalarial medicine used in the two study areas is statistically significant (P<0.001). A statistically significant difference was also observed in the prevalence of the T76 allele among subjects using ACT in the two study areas (P=0.004).

**Table 2:** Type of antimalarial drug used in the treatment of malaria by subjects harbouring Pfcrt alleles.

	Rural			Urban				Grand Total		
Medication used	n	K76	T76	n	K76	T76	P-value	n	K76	T76
ACT	1	0 (0)	1 (100)	10	4 (40)	6 (60)		11 (100)	4 (36.4)	7 (63.6)
			(7.1)*			(66.7)*	0.00*			(31.8)*
Artesunate	0	0 (0)	0 (0)	3	1 (33.33)	2 (66.7)		3 (100)	1 (33.3)	2 (66.7)
			(0)*			(22.2)*	0.08*			(9.1)*
SP	0	0 (0)	0 (0)	2	1 (50)	1 (50)		2 (100)	1 (50)	1 (50)
			(0)*			(11.1)*	0.23*			(4.6)*
Not known	13	1 (7.7)	12 (92.3)	2	2 (100)	0 (0)		15 (100)	3 (20)	12 (80)
			(92.9)*			(0)*	0.00*			(54.6)*
Total	14	1 (7.1)	13 (92.9)	17	8 (47.06)	9 (52.9)		31 (100)	9 (29.0)	22 (71.0)

n: number examined; ()*: proportions across column; P-value*: probability value between proportions of T76 in the rural area and urban area; ACT: artemisinin combination therapy; SP: sulphadoxine-pyrimethamine.

**Figure 3:**
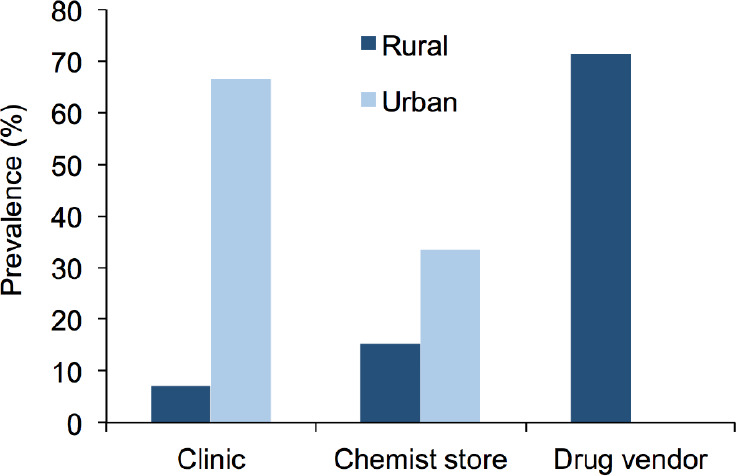
Prevalence (%) of *Pfcrt* T76 mutation and source of antimalarial drug used.

Twenty-two of the 31 subjects using antimalarial medicine had the *Pfcrt* T76 mutant allele. In the rural area, a higher percentage (71.4%) of subjects that had the mutant allele and used antimalarial medicine purchased these from drug vendors, while 15.4% and 7.1% procured antimalarial medicine from chemist stores or clinic, respectively. In the urban area, a higher percentage (66.7% and 33.3%) of subjects that harboured the mutant allele obtained antimalarial medicine from the clinic or chemist store, respectively. However, a significantly higher prevalence of the *Pfcrt* T76 mutant allele was recorded among subjects that sourced antimalarial medicine from drug vendors in the rural area compared to the urban area (P<0.05) (Figure 3).

## 4 Discussion

Information on the prevalence of the mutant *Pfcrt* T76 allele in Ogun State, southwest Nigeria, has been scanty since the withdrawal of CQ in 2005. Previous studies, conducted between 2008 and 2009, reported a prevalence of 97% in Sagamu, Ogun state, and 48% prevalence in Abeokuta, Ogun State [4,13]. The 69.6% prevalence of the *Pfcrt* T76 mutation recorded in this study is considered very high, eight years after the ban on CQ usage in Nigeria and is similar to the result reported by Olukosi *et al.* [16]. This is not surprising because a previous report had shown that CQ remained widely used at the community level even five years after its withdrawal [17]. Results from the present study are unlike the situation reported from other countries like Tanzania, Malawi and Zambia. CQ withdrawal in Tanzania resulted in >90% recovery of susceptibility within ten years of withdrawal [18]. In Zambia and Malawi, a 100% recovery of the *Pfcrt* K76 was achieved 10-13 yrs after the discontinued use of chloroquine.

In our study, the prevalence of chloroquine-resistant malaria parasites was significantly higher in the rural area compared to the urban area. This observation in the rural area is associated with self-prescribed medications, uncontrolled use of herbal medications, persistent drug pressure and the use of substandard antimalarial drugs sourced from unauthorized drug vendors. The majority of subjects that sourced antimalarial medicine from drug vendors lack knowledge of the type of antimalarial drugs sold to them. A previous study already reported that the sale of substandard drugs purchased from drug vendors and incorrect prescription of antimalarial medicine is a common practice in Abeokuta [19]. Inadequate drug exposure through improper dosing and fake drugs accelerate drug resistance development as reported in an earlier study [20]. Although dosing and drug quality was not investigated in our study these factors unknowingly may have contributed to the increased prevalence of the chloroquine drug resistance marker. Uncontrolled use of herbal concoctions prepared from combination of different plants is also common practice in the rural areas [21,22]. However, this common use of herbal medicine is not associated with a reduction in the prevalence of chloroquine-resistant malaria parasites in the rural area.

Although some studies have associated the K76T mutation to severe malaria, while others have found no such association [18], we found no association between the *Pfcrt* T76 mutant allele and disease severity. However, in the urban area we found a significant association between the mutant allele and malaria symptoms. This is so because the clinic is not the first point of call for malaria symptomatic patients in this locality. Reports of previous studies had shown that symptomatic patients practice self-medication either by using herbal concoctions or purchase antimalarial drugs from chemist stores where they are easily accessible for their malaria treatment. Symptomatic patients visit the clinic only when symptoms persist after days of self-medication or severity of disease increases [17,23]. However, we did not investigate whether or not patients practiced self-medication before visiting the clinic.

## 5 Conclusion

We documented factors contributing to the persistent high prevalence of the *Pfcrt* T76 mutant allele, especially in a rural area. These findings have important public health implications especially now that antimalarial drug resistance is a threat to malaria eradication. Hence, it will be important to direct and focus interventions for creating awareness on the importance of using recommended drugs from government-approved sources to lessen the use of less efficacious antimalarials and herbs, especially in rural areas. In addition, government drug regulatory agencies should constantly monitor antimalarial drugs sold by drug vendors/retailers. Continued surveillance of the *Pfcrt* K76T mutation is equally necessary to monitor if resistance will reduce further as has been observed in other African countries.

## References

[1] World Health Organization. (2016). World Malaria Report. http://www.who.int/malaria/publications/world-malaria-report-2016/en/.

[2] Contacos PG, Coatney GR, Lunn JS, Chin W (1965). Resistance to cycloguanil pamoate (CI-501) by falciparum malaria in west Pakistan.. Am. J. Trop. Med. Hyg..

[3] Young MD, Burgess RW (1959). Pyrimethamine resistance in *Plasmodium vivax* malaria.. Bull. World Health Org..

[4] Efunshile M, Runsewe-Abiodun T, Ghebremedhin B, Konig W (2011). Prevalence of the molecular marker of chloroquine resistance (pfcrt 76) in Nigeria 5 years after withdrawal of the drug as first-line antimalarial: A cross-sectional study.. SAJCH.

[5] Carrara VI, Zwang J, Ashley EA, Price RN (2009). Changes in the treatment responses to artesunate-mefloquine on the Northwestern border of Thailand during 13 years of continuous deployment.. PloS ONE.

[6] Dondorp AM, Nosten F, Yi P, Das D (2009). Artemisinin resistance in *Plasmodium falciparum* malaria.. N. Engl. J. Med..

[7] Noedl H, Se Y, Schaecher K, Smith BL (2008). Evidence of artemisinin-resistant malaria in western Cambodia.. N. Engl. J. Med..

[8] Noedl WO, Sem R, Tero T, Chim P (2009). Failure of artesunate-mefloquine combination therapy for uncomplicated *Plasmodium falciparum* malaria in southern Cambodia.. Malar. J..

[9] Anderson TJ, Nair S, Nkhoma S, Williams JT (2010). High heritability of malaria parasite clearance rate indicates a genetic basis for artemisinin resistance in western Cambodia.. J. Infec. Dis..

[10] Laufer MK, Thesing PC, Eddington ND, Masonga R (2006). Return of chloroquine antimalarial efficacy in Malawi.. N. Engl. J. Med..

[11] Umar RA, Hassan SW, Ladan MJ, Nma Jiya M, Abubakar MK, Nataàla U (2007). The association of K76T mutation in *pfcrt* gene and chloroquine treatment failure in uncomplicated *Plasmodium falciparum* malaria in a cohort of Nigerian children.. J. Appl. Sci..

[12] Mwanza S, Joshi S, Nambozi M, Chileshe J (2016). The return of chloroquine‑susceptible *Plasmodium falciparum* malaria in Zambia.. Malar. J..

[13] Olasehinde GI, Ojurongbe OO, Fagade EO, Ruchi S (2014). Detection of molecular markers of antimalarial drug resistance in *Plasmodium falciparum* from South-Western Nigeria.. Covenant J. Phys. Life Sci (CJPL).

[14] Olukosi YA, Iwalokun BA, Magbagbeola OA, Akinwande AI (2005). Pattern of rural-urban acquisition of pfcrt T76 allele among Nigerian children with acute uncomplicated *Plasmodium falciparum* malaria.. Afr. J. Biotechnol..

[15] University of Maryland. Center for Vaccine Development, Malaria Group. PCR and allele-specific restriction analysis (ASRA) of *Pfcrt* codon 76.. http://www.medschool.umaryland.edu/malaria/Protocols.

[16] Olukosi YA, Oyebola MK, Ajibaye O, Orok BA (2014). Persistence of markers of chloroquine resistance among *P. falciparum* isolates recovered from two Nigerian communities.. MalariaWorld J..

[17] Omole MK, Onademuren OT (2010). A survey of antimalarial drug use practices among urban dwellers in Abeokuta, Nigeria.. Afr. J. Biomed. Res..

[18] Afoakwah R, Boampong JN, Egyir-Yawson A, Nwaefuna EK (2014). High prevalence of PfCRT K76T mutation in *Plasmodium falciparum* isolates in Ghana.. Acta Trop..

[19] Idowu OA, Apalara SB, Lasisi AA (2006). Assessment of quality of chloroquine tablets sold by drug vendors in Abeokuta, Nigeria.. Tanzan. Health Res. Bull..

[20] Petersen I, Eastman R, Lanzer M (2011). Drug-resistant malaria: Molecular mechanisms and implications for public health. Federation of European. Biochem. Sci. Lett..

[21] Idowu OA, Soniran OT, Ajana O, Aworinde DO (2010). Ethnobotanical Survey of antimalarial plants used in Ogun State, Southwest Nigeria.. Afr. J. Pharm. Pharmacol..

[22] Tatfeng YM (2011). The attitude of patients towards the treatment of malaria in Edo State, Nigeria.. East Cent. Afr. J. Pharma. Sci..

[23] Idowu OA, Mafiana CF (2007). Malaria in pregnancy: Knowledge, attitude and practices of pregnant women in Abeokuta, Nigeria.. Niger. J. Parasitol..

